# Fabrication of Hierarchical MOF-Derived NiCo_2_S_4_@Mo-Doped Co-LDH Arrays for High-Energy-Density Asymmetric Supercapacitors

**DOI:** 10.3390/nano13192663

**Published:** 2023-09-28

**Authors:** Siyi Cheng, Kang Du, Xiaowu Wang, Yufei Han, Longxiao Li, Guojun Wen

**Affiliations:** School of Mechanical Engineering and Electronic Information, China University of Geosciences, Wuhan 430074, China; chengsiyi@cug.edu.cn (S.C.)

**Keywords:** asymmetric supercapacitors, NiCo_2_S_4_, Co-LDH, cross-linked, Mo-doped, high energy density

## Abstract

The rational fabrication of composite structures made of mixed components has shown great potential for boosting the energy density of supercapacitors. Herein, an elaborate hierarchical MOF-derived NiCo_2_S_4_@Mo-doped Co-LDH arrays hybrid electrode was fabricated through a step-wise method. By leveraging the synergistic effects of a uniform array of NiCo_2_S_4_ nanowires as the core and an MOF-derived porous shell, the NiCo_2_S_4_@Mo-doped Co-LDH hybrid electrode demonstrates an exceptional specific capacitance of 3049.3 F g^−1^ at 1 A g^−1^. Even at a higher current density of 20 A g^−1^, the capacitance remains high at 2458.8 F g^−1^. Moreover, the electrode exhibits remarkable cycling stability, with 91% of the initial capacitance maintained after 10,000 cycles at 10 A g^−1^. Additionally, the as-fabricated asymmetric supercapacitor (ASC) based on the NiCo_2_S_4_@Mo-doped Co-LDH electrode achieves an impressive energy density of 97.5 Wh kg^−1^ at a power density of 835.6 W kg^−1^. These findings provide a promising approach for the development of hybrid-structured electrodes, enabling the realization of high-energy-density asymmetric supercapacitors.

## 1. Introduction

Highly efficient energy storage systems are essential for reducing environmental pollution, geothermal problems, and energy shortage. Among them, supercapacitors have drawn abundant attention due to their exceptional cycle stability, high power density, and fast charge/discharge capabilities [1,2,3]. However, relatively lower energy density than secondary battery systems has restricted their potential for long-term applications [4,5]. The energy density of supercapacitors has been improved by fabricating asymmetric supercapacitors with large voltage windows, as well as developing high-capacitive electrode materials according to E = ½CV^2^ [6,7]. Asymmetric supercapacitors have emerged as a promising solution to bridge the gap between electrical double-layer capacitors (EDLCs) and pseudocapacitors, which are classified based on their charge storage mechanisms. To achieve this, researchers have explored different strategies to integrate materials from both types, aiming to enhance the performance and energy storage capabilities of these devices [8,9,10].

High-specific-capacitance pseudocapacitive material, as an essential part of asymmetric supercapacitors, is crucial for increasing energy density [11]. Recently, pseudocapacitive materials based on hierarchical core-shell structures have been extensively studied due to the adjustable heterostructures, rich redox sites, sufficient electrode/electrolyte contact area, and efficient ion transportation path [12,13,14,15]. Among different materials, NiCo_2_S_4_ is promising for use in supercapacitors because its rich electrochemistry can be easily activated by means to improve the specific capacitance, ion diffusion kinetics, and cyclability. These strategies, particularly those combining NiCo_2_S_4_ with trimetallic oxides and layered double oxides (LDHs) that lead to highly porous core-shell architecture, have demonstrated outstanding performance [16,17,18,19,20]. In our previous work, using NiCo_2_S_4_ as scaffold to construct core-shell structures has been proven effective at increasing the energy density of asymmetric supercapacitors [21,22]. Hence, exploring new strategies for fabricating a NiCo_2_S_4_-based core-shell structure to further enhance supercapacitor performance is promising.

Hydrotalcite-like ionic layered structures, adjustable chemical composition, and high redox activity make layered double hydroxides (LDHs) attractive as electrode materials in supercapacitors [23,24,25]. By integrating LDHs on NiCo_2_S_4_, the high-conductivity core facilitates the electron/ion transport. In contrast, the utilization of a shell with a significantly large specific surface area facilitates efficient ion transport and accelerates the transfer of electrons and ions. In this way, electrochemical performance can be much improved. Among different LDH synthesis methods, using metal organic frameworks (MOFs) as template could improve specific surface areas, facilitate electron transport, and increase the number of electroactive sites, leading to a higher specific capacitance and robust cycle stability [26,27,28,29]. Due to its clearly delineated structure and composition, endowed with traits conducive to viability, the incorporation of secondary metals in the electrode material holds significant potential for achieving the desired properties, opening up new possibilities for future applications. Furthermore, the persistent affinity of substrate molecules towards unsaturated sites, even after the post-treatment process, combined with the presence of extensive cavities, not only enhances reaction kinetics but also preserves rate efficiency [30,31].

Herein, a rational MOF-derived NiCo_2_S_4_@Mo-doped Co-LDH core-shell structure was fabricated through a step-wise method. In particular, NiCo_2_S_4_ nanowires, as the core material, are not only highly pseudocapacitive but also provide conductive support for Mo-doped Co-LDH. The implementation of a core-shell structure significantly enhances the nanomaterial’s specific surface area, and provides protection for NiCo_2_S_4_ during the cycling process. Due to the synergistic effect, this unique structure of NiCo_2_S_4_@Mo-doped Co-LDH delivers extraordinary electrochemical performance. The asymmetric supercapacitor (ASC) device also performed exceptionally well. The remarkable electrochemical properties exhibited by the core-shell NiCo_2_S_4_@Mo-doped Co-LDH, synthesized using MOF as a template, present a promising approach for the development of high-energy-density supercapacitors.

## 2. Materials and Methods

### 2.1. Synthesis of NiCo_2_S_4_ Nanowires

Initially, a carbon cloth measuring 3 × 4 cm^2^ was prepared and subjected to sequential cleaning in acetone, ethanol, and distilled water for a duration of 30 min using ultrasonic treatment. The synthesis of NiCo_2_S_4_ involved the following steps: Urea (0.726 g), CoCl_2_·6H_2_O (1.954 g), and NiCl_2_·6H_2_O (0.951 g) were dissolved in 60 mL of deionized water and stirred magnetically for 1 h. The carbon cloth was then submerged in the solution mentioned earlier and introduced into an autoclave, where it was subjected to a thermal treatment at 120 °C for 6 h. Subsequently, the cloth was allowed to cool naturally. In the final step, the carbon cloth, now loaded with the precursor, was immersed in a 70 mL aqueous solution of Na_2_S_9_·H_2_O (4.8 g) and held at a temperature of 160 °C for a duration of 6 h.

### 2.2. Synthesis of NiCo_2_S_4_@Co-MOF

To synthesize NiCo_2_S_4_@Co-MOF, A (Co(NO_3_)_2_·6H_2_O (0.727 g) in 50 mL of DI water) and B (2-methylimidazole (2-MIM) (1.642 g) in 50 mL of DI water) were prepared in advance. Then, solution B was rapidly added to solution A, and the resulting mixture was vigorously stirred. Following this, the NiCo_2_S_4_ sample was immersed in the mixed solution and allowed to age for 4 h at room temperature. The color of the sample transitioned from black to purple during the aging process. The sample was then washed with methanol and subsequently dried at 60 °C for 12 h to obtain NiCo_2_S_4_@Co-MOF.

### 2.3. Synthesis of NiCo_2_S_4_@ Mo-Doped Co-LDH

In a typical synthesis procedure, a mixture of 20 mL ethanol and 40 mL deionized water was prepared, and 0.3 g of Na_2_MoO_4_·2H_2_O was dissolved in this solution with continuous stirring to achieve homogeneity. Subsequently, the synthesized NiCo_2_S_4_@Co-MOF sample was immersed in this solution and subjected to treatment at 80 °C for 3 h. After cooling, the sample was washed with ethanol and subsequently dried at 60 °C overnight to obtain a hierarchical core-shell nanostructure of NiCo_2_S_4_@Mo-doped Co-LDH.

### 2.4. Material Characterizations

The synthesized samples underwent characterization to determine their phase, morphology, and microstructure. Phase analysis was conducted using a Bruker X-ray diffractometer (XRD), while morphological examination was performed using a NovaNano-450 FEI field emission scanning electron microscope (FESEM). Microstructural analysis was carried out using a JEM-2100 transmission electron microscope (TEM). Additionally, the surface characteristics of the samples were investigated using X-ray photoelectron spectroscopy (XPS) with an Escalab250 instrument.

### 2.5. Electrochemical Measurements

Various techniques were employed to evaluate the electrochemical performance of the electrodes prepared in this study. The electrochemical workstation (PARSTAT-3000A-DX) was utilized to conduct cyclic voltammetry (CV), galvanostatic charge–discharge (GCD), and electrochemical impedance spectroscopy (EIS) in a 3 M KOH electrolyte. To assess the cyclic stability of the electrodes, a battery test system (LAND CT3002A) was employed. In the three-electrode configuration, the working electrode consisted of the prepared samples, the reference electrode was Hg/HgO, and the counter electrode was composed of a Pt plate. The frequency range for EIS measurements spanned from 100 kHz to 0.01 Hz. The electrode capacitance can be determined by analyzing the GCD curve using the following equation:

(1) $$C_{s}=I∆t/m∆V$$

where $C_{s}$ represents the specific capacitance (F g^−1^), I stands for the discharge current (A), t is the discharge time (s), V is the discharge voltage (V), and m is the mass of the active material (g).

### 2.6. Preparation of All-Solid-State Asymmetric Supercapacitor (ASC)

Utilizing the NiCo_2_S_4_@Mo-doped Co-LDH as the positive electrode and activated carbon (AC) as the negative electrode, an ASC device was constructed. Achieving optimal performance necessitates ensuring charge balance between the two electrodes:

(2) $$\frac{m_{+}}{m_{-}}=\frac{C_{S-}∆V_{-}}{C_{S+}∆V_{+}}$$

where m stands for the mass of active material (g), $C_{S}$ is the specific capacitance (F g^−1^), and *V* is the voltage range (V). The specific capacitance of the electrodes could be calculated by the GCD results.

To construct an asymmetric supercapacitor (ASC), the electrodes and filter paper were immersed separately in a PVA-KOH solution for a duration of 15 min. Following this, they were assembled in a sandwich configuration. The electrochemical performance of the ASC device was evaluated by determining the energy density (*E*, Wh kg^−1^) and power density (W kg^−1^) through the utilization of the following equations:

(3) $$E={C\left(∆U\right)}^{2}/7.2$$

(4) P=3600E/∆t

## 3. Results and Discussion

### 3.1. Preparation and Characterizations of the NiCo_2_S_4_@Mo-Doped Co-LDH

**Figure 1:**
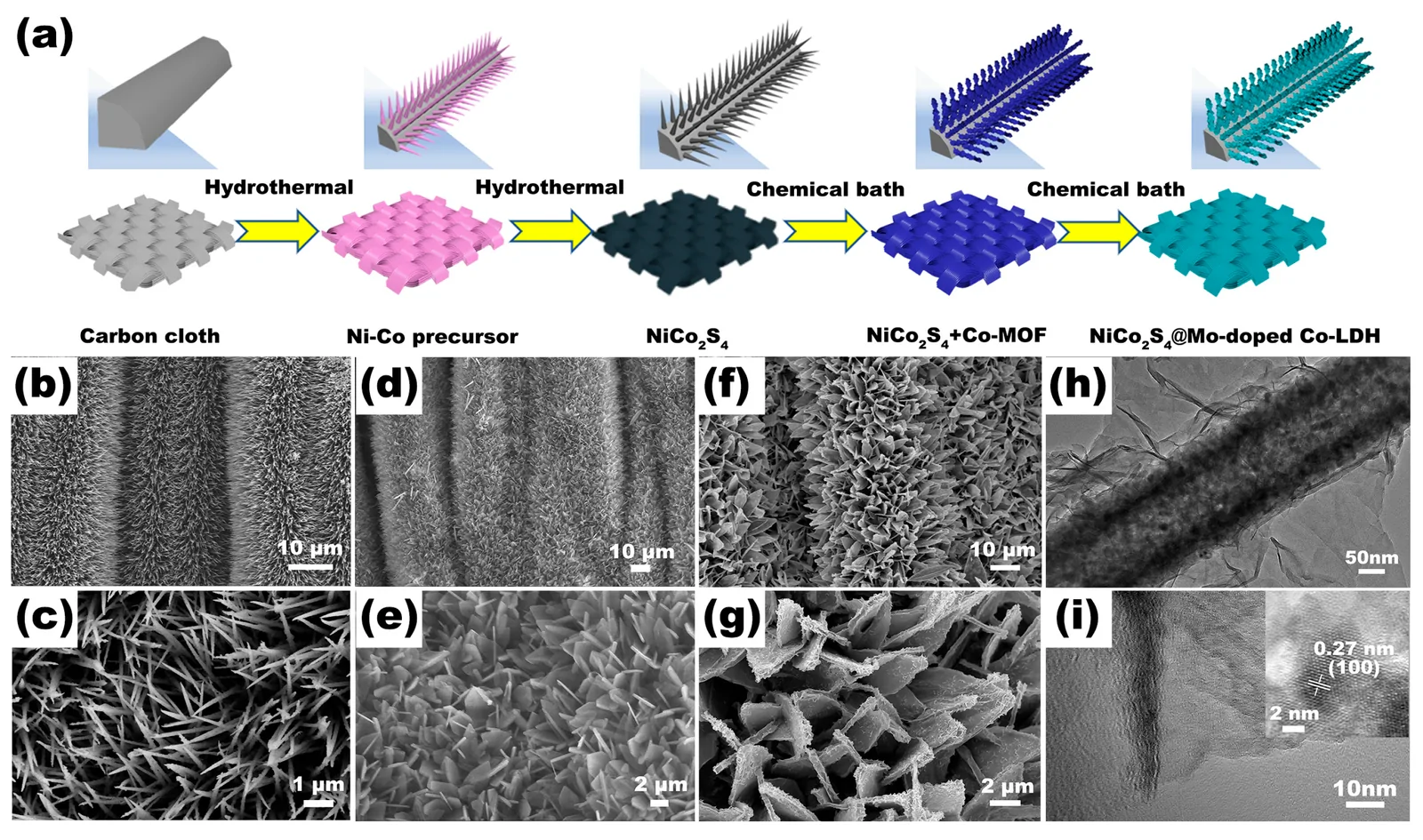
(**a**) Synthesis process of the NiCo_2_S_4_@Mo-doped Co-LDH. Low- and high-resolution SEM images of the (**b**,**c**) NiCo_2_S_4_, (**d**,**e**) NiCo_2_S_4_@Co-MOF, (**f**,**g**) NiCo_2_S_4_@Mo-doped Co-LDH. (**h**,**i**) Low- and high-resolution TEM images of the NiCo_2_S_4_@Mo-doped Co-LDH.

Figure 1a exhibits the synthesis process of the NiCo_2_S_4_@Mo-doped Co-LDH. NiCo_2_S_4_ nanowires were fabricated by a conventional hydrothermal method, which was reported in our previous work. With the initial hydrothermal treatments, the carbon fibers were evenly coated by the NiCo-precursor nanowires (Figure S1). During this process, the NiCo-precursor nanowires were directly grown on carbon by the reaction containing Ni^2+^, Co^2+^, and urea. Urea was used as the surfactant for the assemble of nanowire structures [32]. The surfaces of the NiCo-precursor nanowires are smooth, and the average diameter is about 240 nm. After the sulfuration process, the as-synthesized NiCo_2_S_4_ nanowires were uniformly anchored on carbon fibers (Figure 1b,c), which act as a good scaffold for MOF. Subsequently, with a chemical bath treatment in Co(NO_3_)_2_/2-MIM mixed solution, well-defined ZIF-67 nanosheets (polyhedrons) are coated on the NiCo_2_S_4_ nanowires to form NiCo_2_S_4_@ZIF-67 core-shell structures (Figure 1d,e). Finally, with Na_2_MoO_4_ in ethanol/DI water as etching solution, Mo ions could be immersed into ZIF-67 to release Co^2+^. Co^2+^/Co^3+^ and Mo ions combine with hydroxide ions to generate Mo-doped Co-LDH. The ZIF-67 template was converted into Mo-doped Co-LDH after 3 h of reaction, but the nanosheet profile and the solid bonding with NiCo_2_S_4_ nanowires are left unaltered (Figure 1f,g). Various synthesis timeframes were used to create samples in order to analyze the synthesis mechanism of Mo-doped Co-LDH structures. During brief reaction periods (2 h), the surfaces of NiCo_2_S_4_@Mo-doped Co-LDH exhibited a subtle increase in roughness in comparison to ZIF-67. Notably, no nanoflakes were observed atop the nanosheets, suggesting that the etching process remains incomplete. (Figure S1a,b). With the extension of the synthesis duration to 3 h, the ZIF-67 template remained remarkably unaltered, while the Mo-doped Co-LDH evolved into exquisite 2D nanoflakes, exhibiting elegant growth patterns (Figure S2b,e). The hierarchical arrangement bestowed an expansive active surface, thereby augmenting the capacitance. However, upon prolonging the reaction time to 4 h, the Mo-doped Co-LDH continues its growth, rendering a coarser texture (Figure S2c,f). Consequently, the active sites and electrochemical activity diminished (Figure S4). Among the various samples, the NiCo_2_S_4_@Mo-doped Co-LDH (reaction time = 3 h) showcased a desirable morphology and boasts the highest specific capacitance, rendering it ideal for asymmetric supercapacitors. The microstructure of the specimen was further scrutinized using transmission electron microscopy (TEM). The sample was delicately detached from the carbon cloth substrate. Consistent with the SEM findings, the NiCo_2_S_4_ nanowires were enveloped by Mo-doped Co-LDH nanosheets, resulting in captivating core-shell heterostructures (Figure 1h,i). The lattice D-spacing of 0.27 nm ascribed to (100) crystal plane of α-Co(OH)_2_; this composition will be discussed in the XRD investigations.

**Figure 2:**
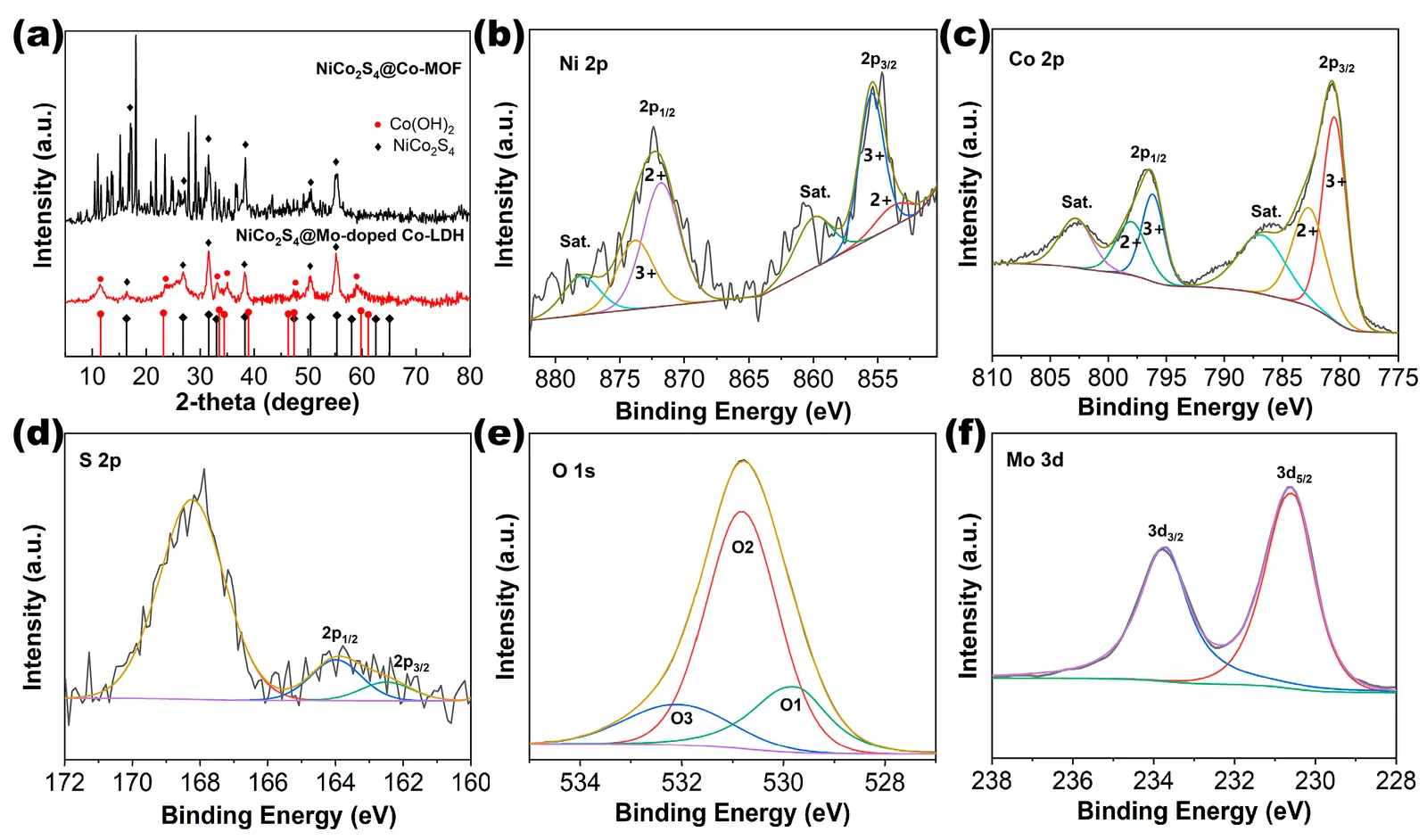
(**a**) XRD patterns of the NiCo_2_S_4_@Co-MOF and the NiCo_2_S_4_@Mo-doped Co-LDH. XPS spectra of the NiCo_2_S_4_@Mo-doped Co-LDH in the (**b**) Ni 2p, (**c**) Co 2p, (**d**) S 2p, (**e**) O 1s, and (**f**) Mo 3d regions.

X-ray diffraction (XRD) was conducted to scrutinize the intricate lattice arrangement and unravel the mystique of the crystal structures of NiCo_2_S_4_@Co-MOF and NiCo_2_S_4_@Mo-doped Co-LDH. Figure S3 exhibits the XRD patterns of NiCo-precursor and NiCo_2_S_4_. The diffraction peaks located at 9.9°, 17.5°, 19.9°, 24.1°, 26.7°, 28.7°, 30.4°, 33.8°, 35.4°, 36.5°, 39.5°, 44.6°, 47.3°, 54.1°, 56.1°, 59.8°, and 62.2° are well indexed with the (020), (001), (111), (220), (121), (300), (221), (040), (301), (231), (050), (340), (060), (142), (412), and (450) planes of Ni_2/3_Co_4/3_(CO_3_)(OH)_2_, respectively [33]. For the NiCo_2_S_4_ nanowires, the diffraction peaks located at 2θ values of 31.6°, 38.3°, 50.5°, and 55.3° could be identified as the (311), (400), (511), and (440) crystal planes (JCPDS No. 20-0782), respectively. As shown in Figure 2a (black line), following the mesmerizing blossoming of the MOF, a symphony of peaks gracefully emerged within the 10–40° range, harmoniously aligning with the resplendent XRD patterns of ZIF-67 [34,35]. The NiCo_2_S_4_ nanowires depict diffraction peaks at 16.3°, 27.1°, 31.6°, 38.3°, 50.5°, 55.2° (marked with circle), which were well correlated with (111), (220), (311), (400), (511), and (440) planes, respectively, clearly showing the cubic spinel crystal of NiCo_2_S_4_ (JCPDS No. 20-0782) [36,37]. The ethereal presence of graphitized carbon gracefully emanated from the carbon cloth, casting a spellbinding allure. This is demonstrated by the broad peaks at around 25°, corresponding to the (002) crystal planes. As for the XRD pattern of NiCo_2_S_4_@Mo-doped Co-LDH (red line of Figure 2a), apart from those NiCo_2_S_4_ planes, peaks at 11.4°, 23.7°, 33.3°, 34.8°, 46.7°, and 59.1° (marked with rhombus) could be ascribed to (003), (006), (100), (102), (108), and (110) planes of α-Co(OH)_2_ (JCPDS No. 46-0605) [38,39]. However, no obvious diffraction peaks related to Mo-based compound present in the XRD pattern of NiCo_2_S_4_@Mo-doped Co-LDH, meaning that the essence of Mo did not orchestrate the birth of a new crystalline realm during the enchanting MOF etching process. Instead, it gracefully infused itself into the very fabric of the MOF-derived Co-LDH, creating a harmonious fusion of elements [40,41].

The captivating elemental composition and chemical valence states of the NiCo_2_S_4_@Mo-doped Co-LDH were investigated using X-ray photoelectron spectroscopy (XPS). The survey spectrum, depicted in Figure S5, revealed the presence of Ni, Co, S, C, Mo, and O elements. Within the Ni 2p spectrum (Figure 2b), a fascinating narrative unfolded, as evidenced by the binding energies of Ni 2p_3/2_ and Ni 2p_1/2_ at 855.1 and 872.9 eV, respectively, indicating the Ni^2+^ valence state. Additionally, the binding energies at 856.3 and 874.1 eV suggested the presence of the Ni^3+^ valence state [42]. Figure 2c unveils a captivating portrayal of the Co 2p spectrum, wherein two distinct peaks emerge with binding energies of 796.3.7 eV and 781.7 eV. These peaks elegantly signify the presence of Co^3+^ species. Another pair of characteristic peaks manifests at binding energies of 792.2 eV and 782.7 eV, distinctly representing Co^2+^. The remarkable disparity of over 15 eV between the binding energies of these doublets elegantly confirms the coexistence of both Co^2+^ and Co^3+^ states. [43,44]. Figure 2d showcases the XPS spectrum of S 2p, revealing two spin-orbit peaks and an accompanying satellite peak. The peak located at 162.5 eV and 163.8 eV can be attributed to S 2p_3/2_ and S 2p_1/2_, while the satellite peak is accredited to surface adsorbed SO_3_^2−^/SO_4_^2−^ species [32,45]. The O 1s XPS spectrum of NiCo_2_S_4_@Mo-doped Co-LDH, illustrated in Figure 2e, showcases a series of peaks associated with the metal–oxygen bond (M-OH), surface-bound OH groups, and chemisorbed water molecules [46]. Furthermore, the Mo 3d spectrum in Figure 2f exhibits two well-defined peaks at 234.0 eV and 231.7 eV, corresponding to Mo 3d_3/2_ and Mo 3d_5/2_, respectively, which can be accurately deconvoluted [47,48]. These peaks unequivocally signify the presence of Mo^6+^ species within the Mo-doped Co-LDH structure. Notably, compared to that of MoO_3_, the Mo 3d spectrum of Mo-doped Co-LDH exhibits a negative shift over 1.0 eV, infesting an augmented electron density on the Mo^6+^ ions resulting from electron abstraction from the Co center [48,49]. This electron transfer leads to an increment in the charge on the Co atom, hence facilitating the redox reaction on the Mo-doped Co-LDH surface.

### 3.2. Electrochemical Measurements of the NiCo_2_S_4_@Mo-Doped Co-LDH

**Figure 3:**
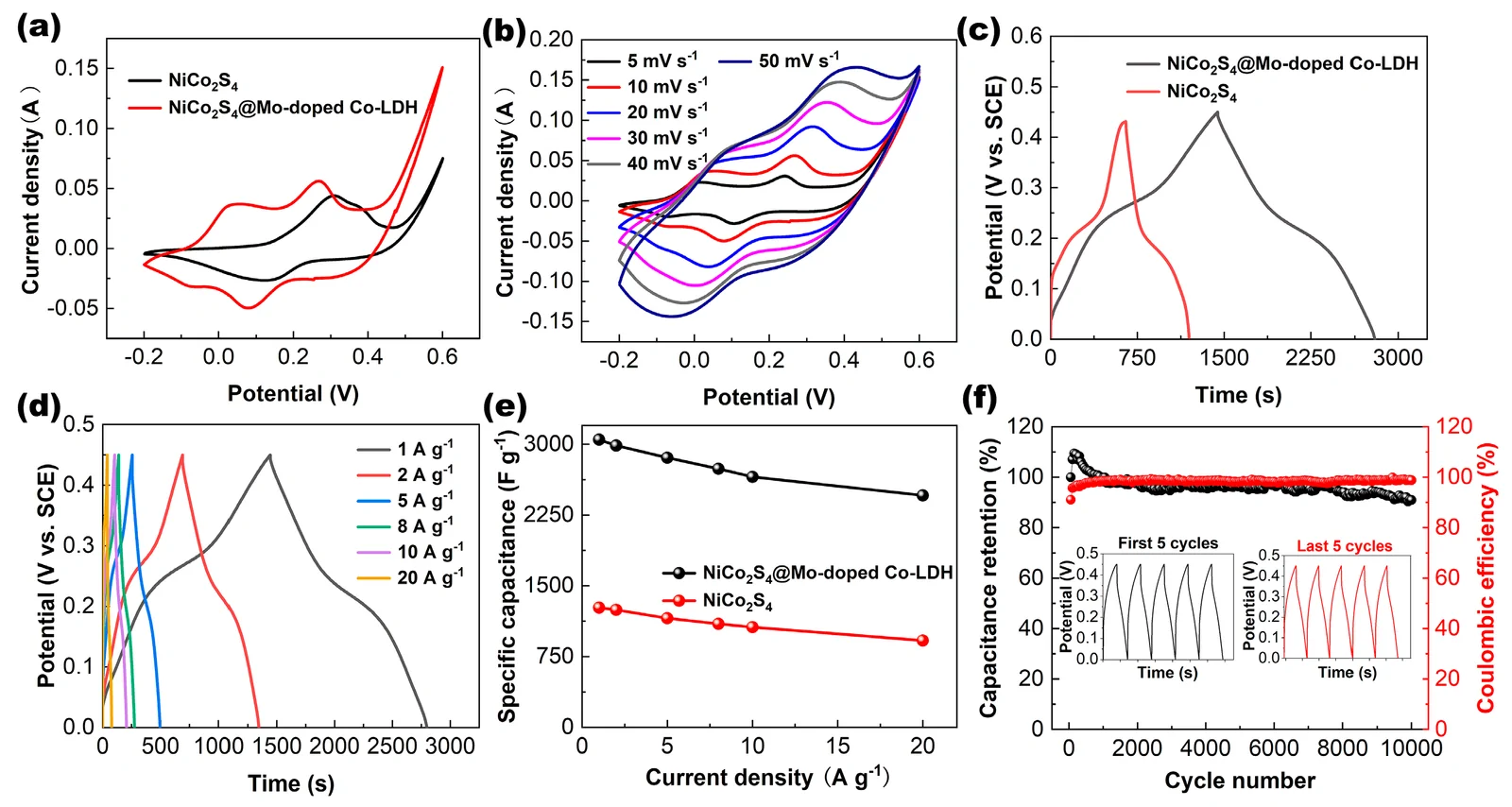
(**a**) CV and (**c**) GCD curves for the NiCo_2_S_4_ and the NiCo_2_S_4_@Mo-doped Co-LDH electrodes recorded at a scan rate of 10 mV s^−1^ and a current density of 1 A g^−1^. (**b**) CV and (**d**) GCD curves of the NiCo_2_S_4_@Mo-doped Co-LDH electrode at various scan rate and current densities. (**e**) Specific capacitance of the NiCo_2_S_4_ and the NiCo_2_S_4_@Mo-doped Co-LDH electrodes at different current densities. (**f**) Cycling performances of the NiCo_2_S_4_@Mo-doped Co-LDH electrode at a current density of 10 A g^−1^.

The electrochemical characteristics of NiCo_2_S_4_ and NiCo_2_S_4_@Mo-doped Co-LDH were assessed using cyclic voltammetry (CV), galvanostatic charge–discharge (GCD) measurements, and electrochemical impedance spectroscopy (EIS) in a three-electrode setup. Figure 3a compares CV plots of the NiCo_2_S_4_ and NiCo_2_S_4_@Mo-doped Co-LDH at 10 mV s^−1^ from −0.2 to 0.6 V. All CV diagrams possess obvious redox peaks, indicating the Faradaic reactions of the electrodes. As for the bare NiCo_2_S_4_, two prominent broad peaks (0.31 and 0.14 V) can be discerned, signifying the occurrence of a redox reaction involving the Co^2+^/^3+^ and Ni^2+^/^3+^ species [50,51]. With the integration of Mo-doped Co-LDH, two anodic peaks (0.02 and 0.28 V) and two cathodic peaks (−0.1 and 0.08 V) appeared, meaning that a more active redox reaction occurs on the composite structure. All the CV curves of these electrodes possess quasi-rectangular profiles, which can be ascribed to the faradaic reaction combined with surface capacitive nature. Moreover, the NiCo_2_S_4_@Mo-doped Co-LDH electrode apparently delivers a larger integral area and peak current than NiCo_2_S_4_, exhibiting an enhanced capacitive performance, the NiCo_2_S_4_@Mo-doped Co-LDH electrode demonstrates a synergistic effect between the two active materials. The cyclic voltammetry (CV) curves of the NiCo_2_S_4_@Mo-doped Co-LDH electrode, presented in Figure 3b, were obtained by varying the scan rates (5–50 mV s^−1^) within a potential window of −0.2 to 0.6 V. It is worth mentioning that the current response demonstrates a direct relationship with the scan rate. Additionally, the anodic and cathodic peaks exhibit a notable shift towards more positive and negative potentials, respectively. Remarkably, the overall shape of the curves remains largely unchanged, indicating the exceptional electrochemical reversibility and high-rate capability of the core-shell NiCo_2_S_4_@Mo-doped Co-LDH electrode.

To investigate the charge storage mechanism of the NiCo_2_S_4_@Mo-doped Co-LDH electrode, the CV curves at different scan rates were analyzed by plotting log peak current vs. log scan rates according to power law (*i* = *av^b^*) (Figure S6a), where *i* is the peak current of CV curves (A), *v* is scan rates (mV s^−1^), and *a* and *b* refer to empirical parameters. When *b* value is close to 0.5, it implies the dominating diffusion controlled faradic process; meanwhile, a *b* value near 1 indicates capacitive controlled process. The *b* values of the NiCo_2_S_4_@Mo-doped Co-LDH electrode are 0.75 and 0.71, implying the co-existence of diffusion and capacitive controlled reversible process [52]. Moreover, to study the charge storage contribution mechanisms of the NiCo_2_S_4_@Mo-doped Co-LDH electrode, the capacitive (*K*_1_*v*) and diffusion (*K*_2_*v*^1/2^) contribution can be quantified according to the following equation: *i* = *K*_1_*v* + *K*_2_*v*^1/2^, where *i* and *v* are the current response and scan rates, respectively (Figure S6b). The contribution histogram (Figure S6c) shows that the charge storage mechanism of the NiCo_2_S_4_@Mo-doped Co-LDH is governed by diffusion-controlled reaction at lower scan rates (69% at 5 mV s^−1^). With the intercalation of ions at higher scan rates, the capacitive contribution increases gradually.

To evaluate the capacitive performance of both the NiCo_2_S_4_ and NiCo_2_S_4_@Mo-doped Co-LDH electrodes, galvanostatic charge–discharge (GCD) measurements were performed within a potential range of 0 to 0.45 V at 1 A g^−1^ (Figure 3c). Both electrodes exhibit distinct potential plateaus and symmetrical shapes, indicating their pseudocapacitive nature, which aligns well with the cyclic voltammetry (CV) results. Notably, the NiCo_2_S_4_@Mo-doped Co-LDH electrode demonstrates an extended discharge time compared to the pristine NiCo_2_S_4_ electrode, implying a significantly higher specific capacitance. Figure 3d presents representative GCD plots of the NiCo_2_S_4_@Mo-doped Co-LDH electrode tested from 1 to 20 A g^−1^, within the potential window of 0 to 0.45 V. The maintained shape of the GCD curves, even at a high current density of 20 A g^−1^, highlights the excellent rate performance, consistent with the CV findings. To achieve a comprehensive understanding of the charge storage mechanism of the electrodes, electrochemical impedance spectroscopy (EIS) measurements were performed (Figure S7). The equivalent circuit diagram (inset of Figure S7) consists of four major components, solution resistance (R_s_), charge transfer resistance (R_ct_), double-layer capacitive (CPE), and Warburg impedance (W) [28]. The Nyquist plots exhibit distinct intercepts where the semicircle intersects the Z’ axis in the high-frequency region, signifying the total internal resistance. Moreover, the sloped line observed in the low-frequency region corresponds to the semi-diffusion process occurring within the electrode materials [53]. Importantly, the EIS curve slope for the NiCo_2_S_4_@Mo-doped Co-LDH electrode is found to be greater than that of the NiCo_2_S_4_ electrode, which suggests enhanced ion diffusion and efficient electron transport within the composite structure.

In Figure 3e, the specific capacitance of the NiCo_2_S_4_@Mo-doped Co-LDH electrode was calculated to be 3049.3, 2986.9, 2857.5, 2741.2, 2654.8, and 2458.8 F g^−1^ at 1, 2, 5, 8, 10, and 20 A g^−1^, respectively. These values significantly exceed those of the bare NiCo_2_S_4_ electrode. Remarkably, even with a rapid increase from 1 to 20 A g^−1^, the NiCo_2_S_4_@Mo-doped Co-LDH electrode maintains 80.6% of its initial specific capacitance, demonstrating its exceptional rate capability. Therefore, this material holds great promise for the fabrication of high-energy-density asymmetric supercapacitors. To assess the cycling performance of the NiCo_2_S_4_@Mo-doped Co-LDH electrode, the relationship between capacitance retention and cycle number is presented in Figure 3f. Following an activation process during the initial stage, the specific capacitance exhibits remarkable cycling stability, with a capacitance retention of 90.1% after 10,000 cycles, accompanied by a coulombic efficiency of approximately 100%. In contrast, under the same conditions, the NiCo_2_S_4_ electrode experiences a decrease to 71.7% (Figure S8), highlighting the beneficial effects of integrating Mo-doped Co-LDH on the structural and electrochemical stability of the core-shell architecture.

### 3.3. Electrochemical Measurements of the NiCo_2_S_4_@Mo-Doped Co-LDH//AC Asymmetric Supercapacitor

**Figure 4:**
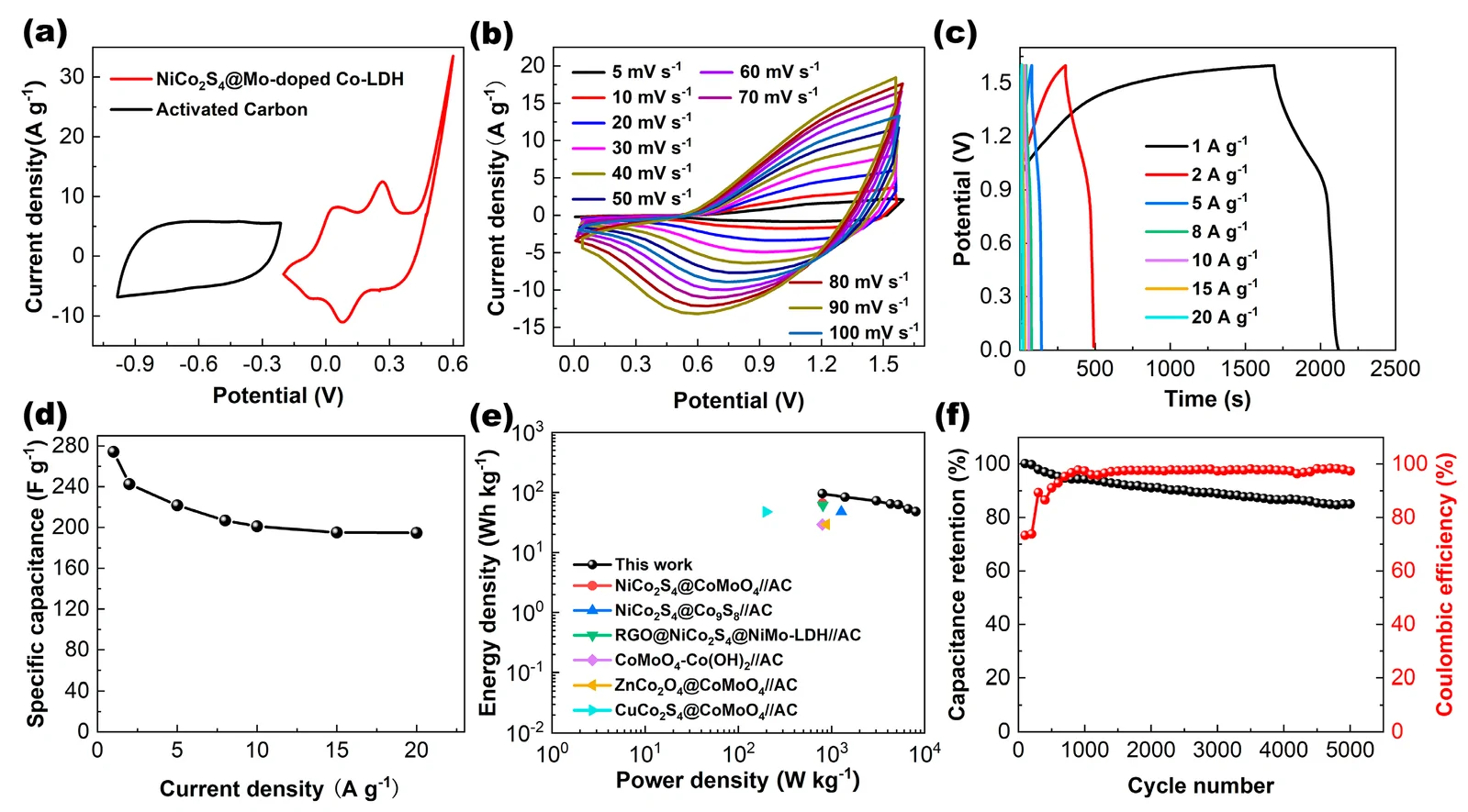
(**a**) CV curves of the AC and the NiCo_2_S_4_@Mo-doped Co-LDH electrodes. (**b**) CV and (**c**) GCD curves of the NiCo_2_S_4_@Mo-doped Co-LDH//AC ASC at various scan rate and current densities. (**d**) Rate capability of the ASC device. (**e**) Ragone plot of the ASC device. (**f**) Cycling stability of the supercapacitor device at 10 A g^−1^.

To evaluate the practical applicability of the synthesized NiCo_2_S_4_@Mo-doped Co-LDH electrode, a flexible all-solid-state asymmetric supercapacitor (ASC) was constructed. The mass ratio of the two electrodes was adjusted to ensure charge balance. The cyclic voltammetry (CV) curves of the activated carbon in the voltage range of −1 to −0.2 V exhibited a rectangular shape, indicating typical electric double-layer capacitance (EDLC) characteristics (Figure 4a). In contrast, the CV curve of the NiCo_2_S_4_@Mo-doped Co-LDH in the voltage range of −0.2 to 0.6 V displayed clear redox peaks, suggesting its pseudocapacitive behavior. This combination of materials effectively extended the voltage window of the device to 1.6 V. Figure S9 demonstrated the CV curves of the ASC device at various potential windows, revealing a gradual broadening of the curves as the potential range increased from 1.0 to 1.6 V. This confirmed the attainment of a 1.6 V potential window. Under this voltage window, Figure 4b illustrated the CV plots of the ASC at different scan rates. As the scan rate increased, the CV curves exhibited a combination of faradaic pseudocapacitive and EDLC characteristics, indicating favorable rate capability and a rapid current-voltage response. The triangular galvanostatic charge–discharge (GCD) plot in Figure 4c, obtained at various current densities, further validated the excellent capacitive behavior of the device. Electrochemical impedance spectroscopy (EIS) measurements (Figure S10) were employed to assess the electrochemical performance. The resulting plot displayed a semicircular shape at high frequencies and a linear shape at low frequencies, indicating outstanding capacitive behavior.

The specific capacitance of the ASC reaches 274.2 F g^−1^ at 1 A g^−1^, and even at a high current density of 20 A g^−1^, the specific capacitance remains at 194.7 F g^−1^, showcasing a remarkable rate capability of 71.1%. This demonstrates the superior capacitive behavior of the NiCo_2_S_4_@Mo-doped Co-LDH//AC ASC device (Figure 4d). The Ragone plot (Figure 4e) shows the relationship between energy density and power density in NiCo_2_S_4_@Mo-doped Co-LDH//AC ASC devices. At a power density of 835.6 W kg^−1^, the ASC device achieves an ultra-high energy density of 97.5 Wh kg^−1^. Even at a high power density of 23,511.7 W kg^−1^, it maintains a maximum energy density of 69.2 Wh kg^−1^.The obtained values are superior to several previously developed core-shell-structure-based ASC devices, including NiCo_2_S_4_@CoMoO_4_//AC (66.6 Wh kg^−1^ at 800 W kg^−1^) [54], NiCo_2_S_4_@Co_9_S_8_//AC (47.7 Wh kg^−1^ at 1275 W kg^−1^) [55], RGO@NiCo_2_S_4_@NiMo-LDH//AC (61.0 Wh kg^−1^ at 808 W kg^−1^) [56], CoMoO_4_-Co(OH)_2_//AC (29.2 Wh kg^−1^ at 800 W kg^−1^) [53], ZnCo_2_O_4_@CoMoO_4_//AC (29.3 Wh kg^−1^ at 884 W kg^−1^) [57], and CuCo_2_S_4_@CoMoO_4_//AC (47.5 Wh kg^−1^ at 199 W kg^−1^) [58]. A comprehensive comparison of NiCo_2_S_4_@Mo-doped Co-LDH with other materials as an asymmetric supercapacitor is presented in Table S1. The charge–discharge cyclic stability under a current density of 10 A g^−1^ is depicted in Figure 4f. After 5000 cycles, the device exhibits a desirable cyclic stability of 85.0%, demonstrating its excellent durability for practical applications.

## 4. Conclusions

In summary, the hierarchical core-shell structure of the NiCo_2_S_4_@Mo-doped Co-LDH electrode, derived from MOF using a step-wise hydrothermal/solvent-thermal method, enhances overall conductivity and facilitates faster ion transport and electron conduction. Integration of MOF-derived Mo-doped Co-LDH further enhances the electrode’s specific capacitance, exhibiting a remarkable value of 3049.3 F g^−1^ at 1 A g^−1^. Additionally, the NiCo_2_S_4_@Mo-doped Co-LDH electrode demonstrates exceptional cyclic stability with 90.1% retention after 10,000 cycles at 10 A g^−1^. The all-solid-state supercapacitor based on this electrode achieves an ultra-high energy density of 97.5 Wh kg^−1^ at a power density of 835.6 W kg^−1^. The rational construction of the hierarchical core-shell structure using MOF as template significantly enhances the electrochemical performance; this characteristic renders it a highly promising contender for the development of future energy storage devices.

## Data Availability

The data presented in this study are available in the article and can be shared upon request.

## Supplementary Materials

The following supporting information can be downloaded at: https://www.mdpi.com/article/10.3390/nano13192663/s1, Figure S1: Low- and high-resolution SEM images of the NiCo-precursor; Figure S2: SEM of the NiCo_2_S_4_@Mo-doped Co-LDH at different synthesis times; Figure S3: XRD patterns of the NiCo-precursor and NiCo_2_S_4_; Figure S4: Comparison of GCD (a) and EIS (b) results of the NiCo_2_S_4_@Mo-doped Co-LDH at different synthesis times; Figure S5: Survey XPS spectra of the NiCo_2_S4@Mo-doped Co-LDH; Figure S6: (a) Linear variation of log peak current and log scan rates. (b) Linear relation of i/v vs. v. (c) Diffusion contribution and capacitive contribution at various scan rates; Figure S7: EIS results of the NiCo_2_S_4_ and NiCo_2_S_4_@Mo-doped Co-LDH; Figure S8: Cycling performance of the NiCo_2_S_4_ electrode at 10 A g^−1^; Figure S9: CV curves of the NiCo_2_S_4_@Mo-doped Co-LDH//AC ASC at various voltage window; Figure S10: EIS curve of the NiCo_2_S_4_@Mo-doped Co-LDH//AC ASC device; Table S1: Comparison of NiCo_2_S_4_@Mo-doped Co-LDH with other materials. Refs. [59,60,61,62,63] are cited in Supplementary Materials.
