# Supplementary material for: Fabrication of Hierarchical MOF-Derived NiCo2S4@Mo-Doped Co-LDH Arrays for High-Energy-Density Asymmetric Supercapacitors
Source: Nanomaterials (Basel). 2023 Sep 28;13(19):2663. doi: 10.3390/nano13192663 (PMC10574694; doi:10.3390/nano13192663)
Supplement: Supplementary file 1 [file nanomaterials-13-02663-s001.zip › nanomaterials-2621185-supplementary.pdf]

Supporting Information

Fabrication of Hierarchical MOF-Derived $NiCo_2S_4$@Mo-Doped Co-LDH Arrays for High-Energy-Density Asymmetric Supercapacitors

Siyi Cheng, Kang Du, Xiaowu Wang, Yufei Han, Longxiao Li and Guojun Wen *

School of Mechanical Engineering and Electronic Information, China University of Geosciences, Wuhan 430074, China; chengsiyi@cug.edu.cn (S.C.)
* Correspondence: wenguojun@cug.edu.cn; Tel.: +86-27-67883421

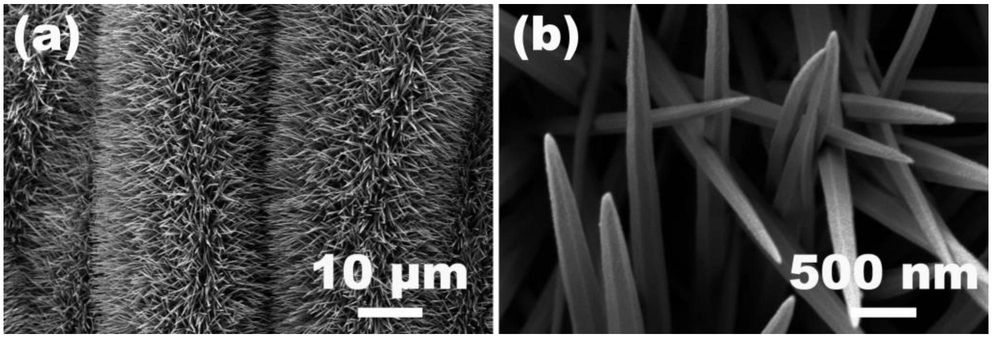

Figure S1. Low and high-resolution SEM images of the NiCo-precursor.

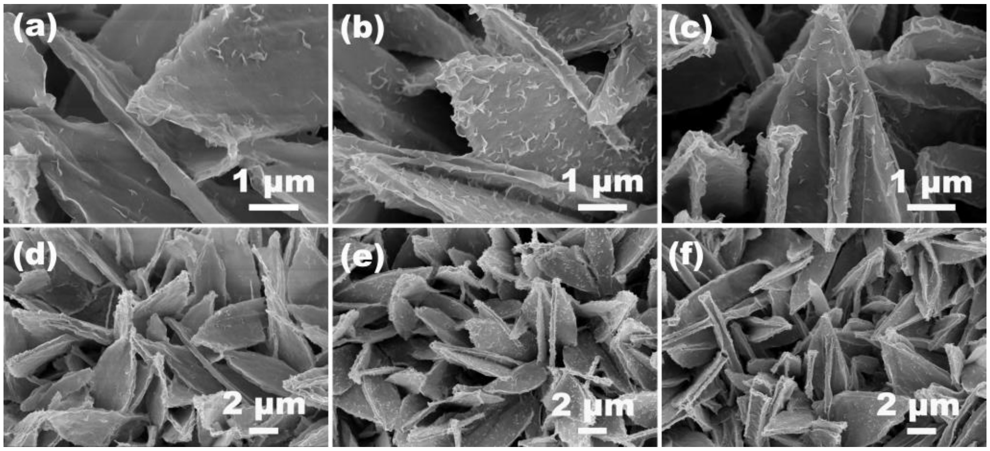

Figure S2. SEM of the $NiCo_2S_4$@Mo-doped Co-LDH at different synthesis time.

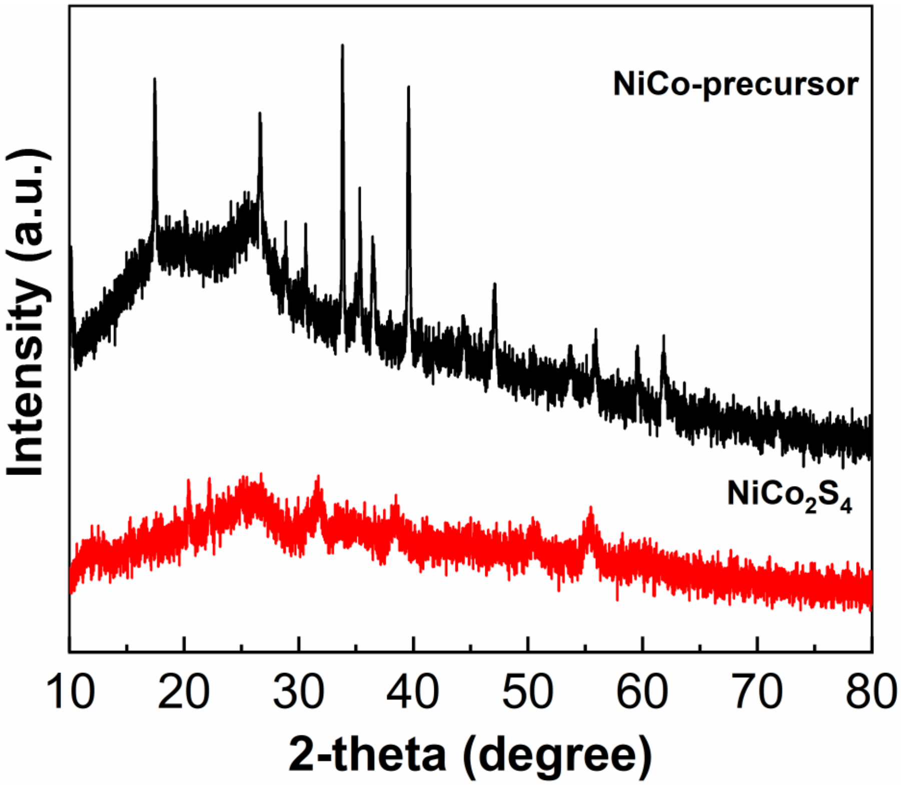

Figure S3. XRD patterns of the NiCo-precursor and $NiCo_2S_4$.

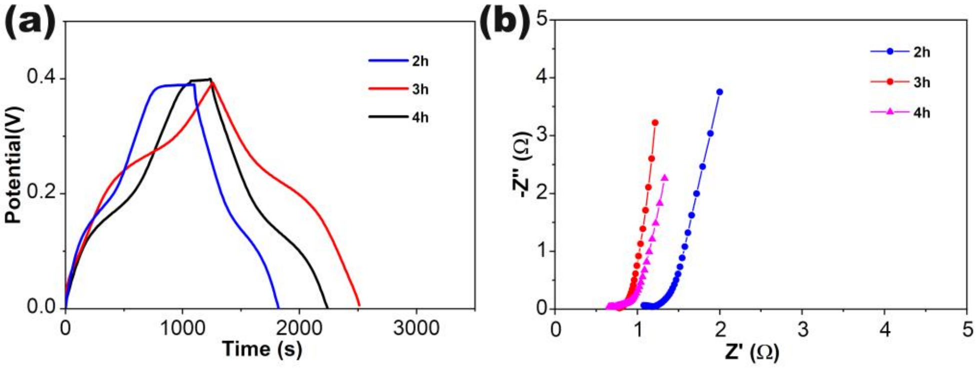

Figure S4. Comparison of GCD (a) and EIS (b) results of the $NiCo_2S_4$@Mo-doped Co-LDH at different synthesis time.

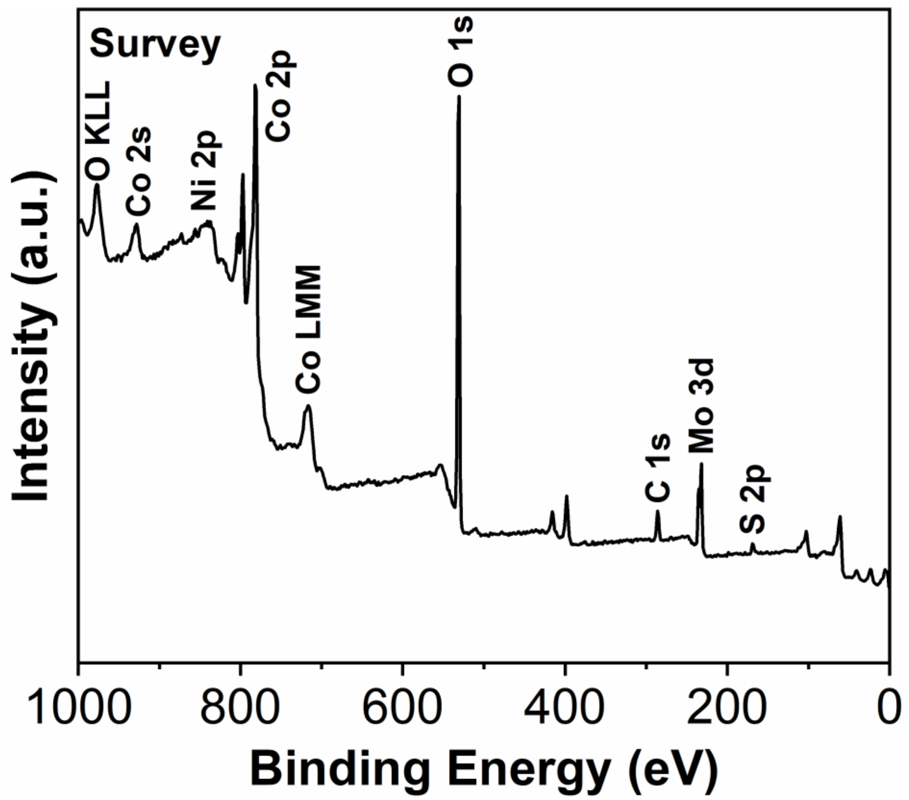

Figure S5. Survey XPS spectra of the $NiCo_2S_4$@Mo-doped Co-LDH.

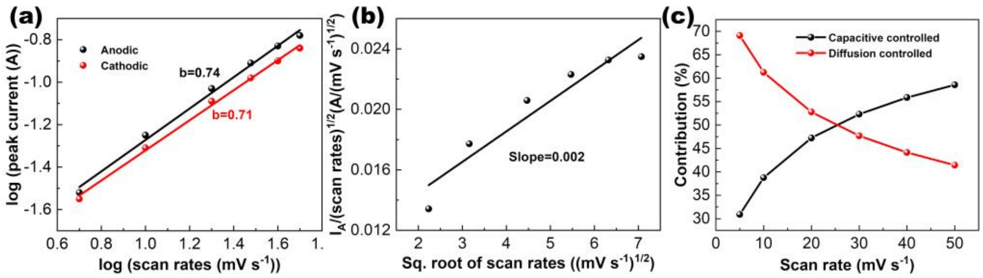

Figure S6. (a) Linear variation of log peak current and log scan rates. (b) Linear relation of i/v vs v. (c) Diffusion contribution and capacitive contribution at various scan rates.

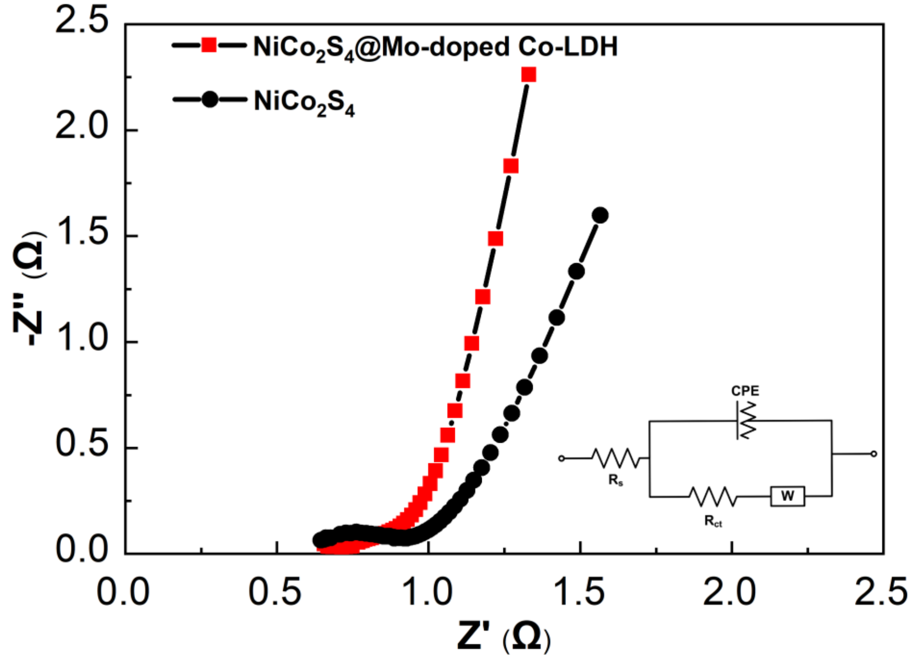

Figure S7. EIS results of the $NiCo_2S_4$ and $NiCo_2S_4$@Mo-doped Co-LDH. Inset: the corresponding equivalent circuit.

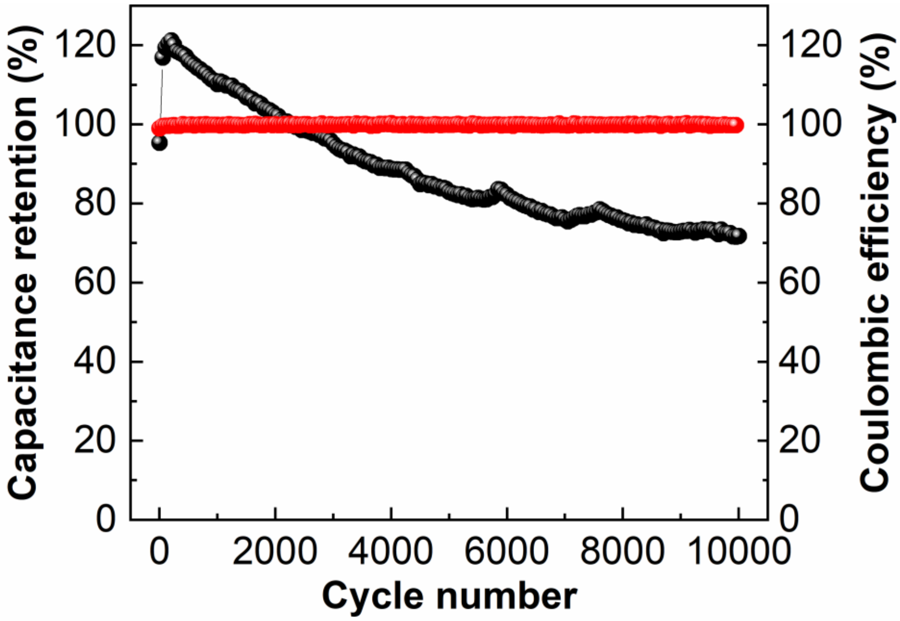

Figure S8. Cycling performance of the $NiCo_2S_4$ electrode at 10 A g^{-1}.

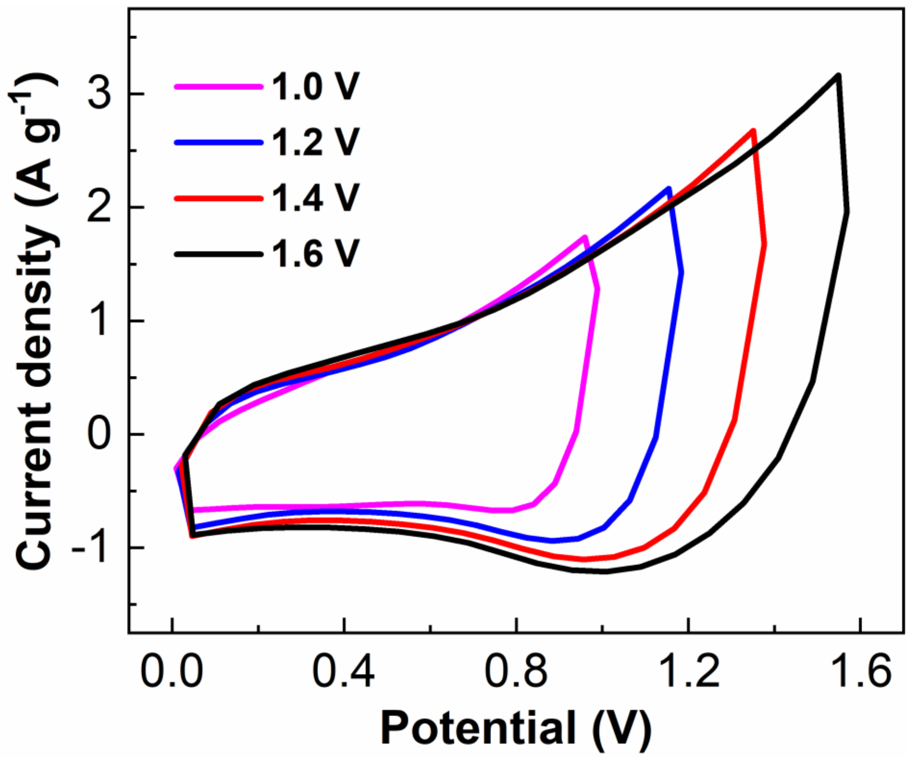

Figure S9. CV curves of the $NiCo_2S_4$@Mo-doped Co-LDH//AC ASC at various voltage window.

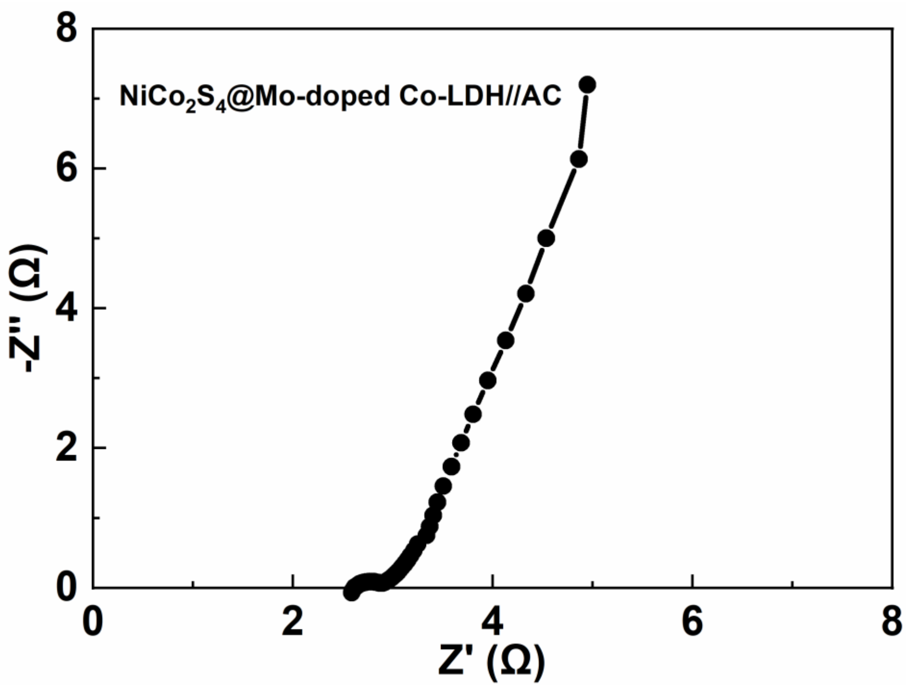

Figure S10. EIS curve of the $NiCo_2S_4$@Mo-doped Co-LDH//AC ASC device.

Table S1. Comparison of $NiCo_2S_4$@Mo-doped Co-LDH with other materials.

Materials	Current density	Specific capacitance	Energy density	Power density	Ref
$NiCo_2S_4$@$CoMoO_4$//AC	1 A g^{-1}	2118.8 F g^{-1}	66.6 Wh kg^{-1}	800 W kg^{-1}	1
$NiCo_2S_4$/Co_9S_8 HSs//AC	1 A g^{-1}	2180 F g^{-1}	96.5 Wh kg^{-1}	800 W kg^{-1}	2
$NiCo_2S_4$@Co_9S_8//AC	1 A g^{-1}	2390.2 F g^{-1}	47.7 Wh kg^{-1}	1275 W kg^{-1}	3
$CoMoO_4$-$Co(OH)_2$//AC	1 A g^{-1}	2028 F g^{-1}	61 Wh kg^{-1}	800 W kg^{-1}	4
ZnS/$NiCo_2S_4$/Co_8S_9 nanotube	1 A g^{-1}	1618.1 F g^{-1}	66 Wh kg^{-1}	750 W kg^{-1}	5
RGO@$NiCo_2S_4$@NiMo-LDH	1 A g^{-1}	1346 F g^{-1}	59.38 Wh kg^{-1}	808.19W kg^{-1}	6
$NiCo_2S_4$@$NiMoO_4$	5mA/cm^2	2006 F g^{-1}	12.6 Wh kg^{-1}	1158W kg^{-1}	7
$CuCo_2S_4$@ $CoMoO_4$//AC	1mA/cm^2	2058 F g^{-1}	45.73 Wh kg^{-1}	198.8 W kg^{-1}	8
CoNi-LDH/$NiCo_2S_4$/RGO//AC	1A g^{-1}	1846.66 F g^{-1}	28.8 Wh kg^{-1}	800 W kg^{-1}	9
$NiCo_2S_4$@MoS_2//AC	3mA/cm^2	719.32 F g^{-1}	48.63 Wh kg^{-1}	250.1 W kg^{-1}	10
$ZnCo_2O_4$@ CoMoO4//AC	10mA/cm^2	1096.08 C g^{-1}	29.24 Wh kg^{-1}	884.57 W kg^{-1}	11
$NiCo_2S_4$@Mo-doped Co-LDH//AC	1 A g^{-1}	3052.88 Fg^{-1}	95.56 Wh kg^{-1}	797.93 W kg^{-1}	This work

References

[1] Zhao, Yunhe, Xinyi He, Rongrong Chen, Qi Liu, Jingyuan Liu, Dalei Song, Hongsen Zhang et al. "Hierarchical $NiCo_2S_4$@ $CoMoO_4$ core-shell heterostructures nanowire arrays as advanced electrodes for flexible all-solid-state asymmetric supercapacitors." Applied Surface Science 453 (2018): 73-82.

[2] Han, Xinru, Qun Chen, Hong Zhang, Yonghong Ni, and Li Zhang. "Template synthesis of $NiCo_2S_4/Co_9S_8$ hollow spheres for high-performance asymmetric supercapacitors." Chemical Engineering Journal 368 (2019): 513-524.

[3] Jia, Hong, Jie Wang, Wenwen Fu, Junhua Hu, and Yu Liu. "In-situ MOFs-derived hollow Co_9S_8 polyhedron welding on the top of $MnCo_2S_4$ nanoneedles for high performance hybrid supercapacitors." Chemical Engineering Journal 391 (2020): 123541.

[4] Zhao, Yunhe, Hongxing Dong, Xinyi He, Jing Yu, Rongrong Chen, Qi Liu, Jingyuan Liu, Hongsen Zhang, Jia Yu, and Jun Wang. "Carbon cloth modified with metal-organic framework derived CC@$CoMoO_4$-$Co(OH)_2$ nanosheets array as a flexible energy-storage material." ChemElectroChem 6, no. 13 (2019): 3355-3366.

[5] Sui, Yanwei, Yuanming Zhang, Haihua Hu, Qin Xu, Fei Yang, and Zhaosheng Li. "High energy density asymmetric supercapacitor based $ZnS/NiCo_2S_4/Co_9S_8$ nanotube composites materials." Advanced Materials Interfaces 5, no. 12 (2018): 1800018.

[6] Cheng, Cheng, Yongjin Zou, Fen Xu, Cuili Xiang, Qingli Sui, Jian Zhang, Lixian Sun, and Zhenming Chen. "Ultrathin graphene@ $NiCo_2S_4$@ Ni-Mo layered double hydroxide with a 3D hierarchical flowers structure as a high performance positive electrode for hybrid supercapacitor." Journal of Energy Storage 52 (2022): 105049.

[7] Zhang, Yan, Jie Xu, Yayun Zheng, Yingjiu Zhang, Xing Hu, and Tingting Xu. "$NiCo_2S_4$@ $NiMoO_4$ core-shell heterostructure nanotube arrays grown on Ni foam as a binder-free electrode displayed high electrochemical performance with high capacity." Nanoscale research letters 12 (2017): 1-9.

[8] Mao, Xiaoqi, Ying Wang, Cuili Xiang, Dan Zhan, Huanzhi Zhang, Erhu Yan, Fen Xu et al. "Core-shell structured $CuCo_2S_4$@ $CoMoO_4$ nanorods for advanced electrode materials." Journal of Alloys and Compounds 844 (2020): 156133.

[9] Guo, Yaning, Chen Hao, Xiaokun Wang, Ying Yang, Xiaohong Wang, Jingbo Wu, and Yutang Shen. "Facile fabrication of CoNi-Layered Double Hydroxide/$NiCo_2S_4$/Reduced Graphene Oxide composites by in situ hydrothermal growth strategy for supercapacitor performance." Ceramics International 48, no. 12 (2022): 17644-17653.

[10] Tang, Yu, Xilong Liu, Yang Chen, Leiyun Han, Dianfeng Dai, Liangyu Liu, Chongtai Wang, Yingjie Hua, Xudong Zhao, and Xiaoyang Liu. "$NiCo_2S_4$@MoS_2 core/shell nanorod arrays for fabrication of high-performance asymmetric supercapacitors with high mass loading." Journal of Energy Storage 51 (2022): 104518.

[11] Yu, Deyang, Yifei Teng, Xilong Liu, and Xiaoyang Liu. "A high-performance electrode based on the $ZnCo_2O_4$@$CoMoO_4$ core-shell nanosheet arrays on nickel foam and their application in battery-supercapacitor hybrid device." Electrochimica Acta 347 (2020): 136278.
